# Fast Track Diagnostic Tools for Clinical Management of Sepsis: Paradigm Shift from Conventional to Advanced Methods

**DOI:** 10.3390/diagnostics13020277

**Published:** 2023-01-11

**Authors:** Ena Gupta, Juhi Saxena, Sanni Kumar, Umang Sharma, Saundarya Rastogi, Vijay Kumar Srivastava, Sanket Kaushik, Anupam Jyoti

**Affiliations:** 1 Amity Institute of Biotechnology, Amity University Rajasthan, Amity Education Valley, Kant Kalwar, NH-11C, Jaipur Delhi Highway, Jaipur 303002, India; 2 Department of Biotechnology, University Institute of Biotechnology, Chandigarh University, Sahibzada Ajit Singh Nagar 140413, India

**Keywords:** antimicrobial resistance, sepsis, early diagnosis, conventional methods, modern methods, advanced methods

## Abstract

Sepsis is one of the deadliest disorders in the new century due to specific limitations in early and differential diagnosis. Moreover, antimicrobial resistance (AMR) is becoming the dominant threat to human health globally. The only way to encounter the spread and emergence of AMR is through the active detection and identification of the pathogen along with the quantification of resistance. For better management of such disease, there is an essential requirement to approach many suitable diagnostic techniques for the proper administration of antibiotics and elimination of these infectious diseases. The current method employed for the diagnosis of sepsis relies on the conventional culture of blood suspected infection. However, this method is more time consuming and generates results that are false negative in the case of antibiotic pretreated samples as well as slow-growing microbes. In comparison to the conventional method, modern methods are capable of analyzing blood samples, obtaining accurate results from the suspicious patient of sepsis, and giving all the necessary information to identify the pathogens as well as AMR in a short period. The present review is intended to highlight the culture shift from conventional to modern and advanced technologies including their limitations for the proper and prompt diagnosing of bloodstream infections and AMR detection.

## 1. Introduction

Sepsis is one of the most ambiguous disorders in medicine due to its early onset. Previously, sepsis was thought to be the process through which flesh decomposes, swamps acquire a putrid odour, and wounds deteriorate [1]. Later, it was renamed as systemic infection, which is commonly referred to as “blood poisoning” and was acknowledged as a result of pathogenic organisms growing within the circulation and evading the host’s immune system. As a result, it was established that the pathogen, not the host, is the perpetrator in the pathophysiology of sepsis [2]. Pathogens interact in the host immune system during infection, triggering a downstream inflammatory cascade including cytokines and other mediators, eventually producing immunosuppression, which leads to various types of organ failure and subsequent clinical degeneration [3].

Originally, sepsis was thought to be the result of internal organs rotting or decaying, with sepsis being defined as a result of the host’s systemic inflammatory response syndrome (SIRS) to infection, severe sepsis (sepsis associated with organ dysfunction, hyperfusion, or hypotension), and septic shock (sepsis induced hypotension that persists despite adequate fluid resuscitation) [4]. However, due to a lack of effective antimicrobials and supportive treatment, the definition of sepsis has evolved with time, preventing patients with sepsis from living long enough to be analyzed or they acquire a sequel of organ failure. As a result, the American College of Chest Physicians (ACCP) and the Society of Critical Care Medicine (SCCM) announced SIRS and published new agreements as a definition of sepsis and associated medical criteria (Sepsis-3) with deadly organ dysfunction caused by a dysregulated host response to an infection, which can be accepted as a shift in complete Sequential Organ Failure Assessment (SOFA) score points ≥2 subsequent toward the disease [3,4,5]. The guidelines of the SOFA score were presumed to be zero in the patients having no infection or organ failure whereas a SOFA score ≥2 mirrors a general fatality danger of approx. 10% in a common emergency population with suspected infection [6]. This novel and advanced definition focus on the power of the non-homeostatic response of the host to infection, the potential fatality of the infection that is impressively more than a direct disease, and the requirement for dire acknowledgement. As depicted later, even an unpredictable level of organ dysfunction when it was suspected earlier is related to an in-emergency mortality rate of nearly 10%.

According to the World Health Organization (WHO), in hospitals, sepsis is not just the costliest condition to treat as an additional main source of death, but certain reports have assessed a great many cases at a greater expense and death rates influencing more than 30 million individuals worldwide, thus steadily prompting 6 million deaths with rates of mortality between 20% and 50% [7]. The weight of sepsis is probably most noteworthy in low-income countries [8]. A reported 31.5 million people have are diagnosed with sepsis annually around the world, of which, 19.4 million individuals suffer from severe sepsis while 5.3 million individuals experience death, 3 million cases are estimated in neonates worldwide annually and there are 1.2 million cases in children, with a death rate of 11–19% [9], as well as 75,000 annual deaths in females due to puerperal sepsis globally [5]. Additionally, as per the ongoing safety information of Centers for Disease Control and Prevention (CDC) reports, sepsis inpatient admissions stay high for septic shock, roughly 60%; for severe sepsis, around 36%; for sepsis credited to a particular creature, roughly 31%; and for unknown sepsis, roughly 27% [10]. The latest in-hospital mortality estimates for sepsis patients has decreased from 28% to 18% [11]. Patients with severe sepsis admitted to the ICU increased from 7.2% to 11.1% and hospital mortality in severe sepsis decreased from 35% to 18%, according to a recent study of prospectively collected data from more than 90% of all intensive care unit (ICU) hospitalizations, confirming these trends in both incidence and mortality. Finally, the best epidemiologic evidence shows that severe sepsis is becoming more common while simultaneously becoming less fatal [12].

Because of its narrow window, early and systemic diagnosis of sepsis is a crucial task which may deviate towards shock, organ failure or even death. Delay in every hour in providing the correct treatment results in a decline in the survival rate of sepsis patients by 7.6% [13]. To combat this, non-specific broad-spectrum antibiotics are administered immediately in suspected cases which results not only in poor patient outcomes but also in the development of multi-drug resistance (MDR) [14,15]. Hence, the diagnostic method should be rapid and applicable to detect pathogens along with drug resistance (preferably within 3–5 h of patient admission) [16,17]. It should be capable enough to diagnose polymicrobial infections along with unknown and emerging pathogens. Furthermore, the results of the diagnosis should be able to provide appropriate decisions and antibiotic stewardship within a key time window of hours in order to limit morbidity and death [14]. The procedures that are sensitive, specific and quick for identifying the pathogen are therefore the major operational instruments for critical care units [18,19].

**Figure 1:**
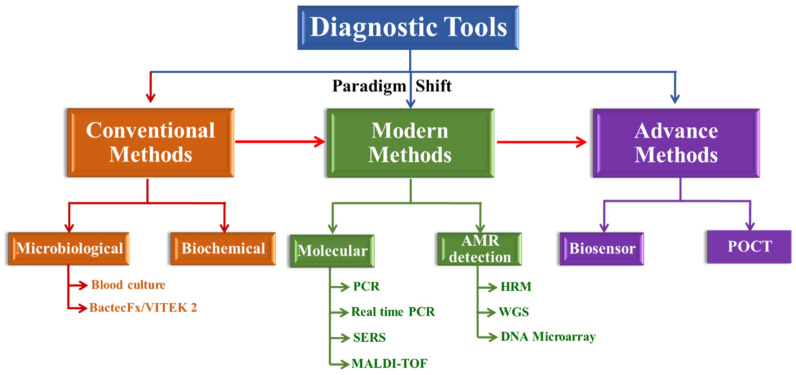
Schematic overview of the diagnostic methods. Conventional methods use the selected cultures and medium in traditional approaches and detect basic aspects of bacterial identification. Molecular detection uses molecular-based (DNA and RNA) approaches to identify resistance genes, as well as mutations in and expression of these genes, or their genomic signature.

The current review discusses the present status of conventional, modern and advanced methods along with their advantages and disadvantages in the deep specialized, translational and application-related scope (Figure 1). The authors searched PubMed and Google Scholar applying the following keywords: sepsis, early diagnosis, conventional as well as modern methods and cited the papers from the year 2018 to 2022. Many case reports and series, as well as retrospective and prospective investigations, systematic reviews and meta-analyses, clinical recommendations and other narrative reviews, have been included by the authors. The search for literature has been limited to research in English exclusively. Over 100 publications were disclosed in the initial literature search. The authors evaluated all the relevant publications and assessed which research to include, with their conventional and modern techniques, including WHO and CDC clinical guidelines, in the review focused on an early identification of the condition.

## 2. Conventional Methods for Management of Sepsis

### 2.1. Microbiological Methods

The detection of pathogens using these techniques is based on the apparent growth of microorganisms on a suitable culture media (solid agar and broth). There are two different methods and they have been discussed in detail in the section below.

#### 2.1.1. Identification through Blood Culture/Gram Staining

For detection as well as identification of causative pathogens, sampling of blood/urine/lavage from patients, their routine culture followed by the Gram staining test remains the gold standard method [20,21,22]. To enhance the diagnosis, a minimum requirement of blood samples collected aseptically is 0.5–1 mL. Preferably, blood is drawn for blood cultures from two distinct venipunctures sites. One is the central venous catheters, which allows blood to be obtained concurrently from a peripheral and a vascular catheter, allows for faster detection of peripheral bacteremia vs. catheter-related bloodstream infections and appropriate dosing for clinical treatment [23]. Since some microbes can only be identified at the collection site and not in the blood, continuous monitoring of the collection sites is necessary when a positive culture is detected, which facilitates the further processing for pathogen identification in proper sepsis assessment [24].

These are the confirmatory tests to detect the potentiality of microbes in the given sample [25]. Comparatively, gram staining analysis is rapid (<15 min), economical, and provides information about the categorization of the infectious microbe as either Gram-positive, Gram-negative, Gram variable or Gram indeterminate [26,27].

#### 2.1.2. Identification through Bactec Fx/VITEK 2

This automated system is built on sensors that detect any change in pressure within the blood culture bottle or track the CO_2_ emitted by actively metabolizing species. Gram-negative microbes take 14 to 24 h in blood culture bottles to detect microbial growth, while Gram-positive bacteria take 24 to 48 h. It is now feasible to eliminate enough pathogenic load for direct identification in Bactec FX without first growing on an agar plate until a blood culture bottle has been marked positive and is suitable for traditional diagnosis [28,29]. Blood culture bottles are prepared, checked and incubated for 5 days at 35 °C with shaking agitation every 10 min in the instrument. By spreading 0.1 mL of consecutive 10-fold dilutions on a blood agar plate, the quantitative plate count technique is used to determine microbial fixation. They are then tested after overnight incubation at 350 °C on plates containing the colonies [30].

The following are the three main issues with using this culture-based method: Firstly, the conventional microbiological tests require 5 days to detect and identify the pathogens involved in sepsis. As a result, the procedure does not provide results promptly, which may be a significant source of anxiety for patients not having the infection. Secondly, the blood culture/Gram stains analysis has a lower sensitivity. Only 30% to 60% of the overall positive result of sensitivity has been estimated despite using it in the proper analytical manner, standardized procedures and accurate collection of amounts of blood sample [28,29,30,31]. These recommended false-negative results which range from 40% to 70% might be owing to a scarcity of particular microbial species that flourish in laboratory culture medium, or distantly similar microbial strains [32,33]. Another reason for the false-negative result is self-medication and administration of antibiotics to the patients before the sampling for diagnosis [34]. Thirdly, there is the possibility of false positives due to non-adherence with the sterile condition during sample processing. As a consequence, patients receive antibiotics for those bacteria of which they are not infected. This misuse has led to the prolonged exposure of antibiotics, resulting in allergic reactions, toxicity, development of MDR, a long stay of patients in hospitals and hence increased medical costs [34,35,36]. As a result, certain points should be proposed for laboratory experts, concerned bodies and other stakeholders to improve the laboratory service by applying and using refined and emerging technology to classify and assess sensitivity to drugs for various microorganisms. However, there is a need for more intensive interventional trials to assess the effect of these technologies on sepsis treatment in clinical practice.

### 2.2. Biochemical Test

Biochemical tests including the mannitol test, citrate tests, triple sugar iron (TSI) test, indole test, methyl red test and enzymatic tests such as oxidase tests, urease tests and coagulase tests are used to distinguish pathogenic organisms that depend on the various diverging biochemical processes of different bacteria. For the identification of extracellular and intracellular bacterial enzymes, biochemical tests are used. Hence, they are distinguished based on biochemical activities [37].

## 3. Modern Methods for Management of Sepsis

There is a paradigm shift from conventional culture and biochemical-based detection of pathogens to modern techniques including molecular as well as emerging methods which rely on detection at the strain level in much less time (20 min to 3 h). System biology has been thrust into the spotlight in molecular research due to technological advances and the knowledge provided by the human genome project, which has accelerated multiple methods that may not only produce an expanded understanding of complicated sepsis pathophysiology but can also articulate undetermined methodologies [38,39]. Reviewed here are the modern advancements in early detection, including inventive high-throughput approaches.

### 3.1. Implementing Molecular Detection for the Identification of Pathogens

Molecular detection using PCR depends upon the amplification of the pathogen’s target nucleic acid region using gene-specific primers or probes. For the identification of different pathogens, a variety of molecular targets are used [40,41,42,43].

#### 3.1.1. PCR

**Figure 2:**
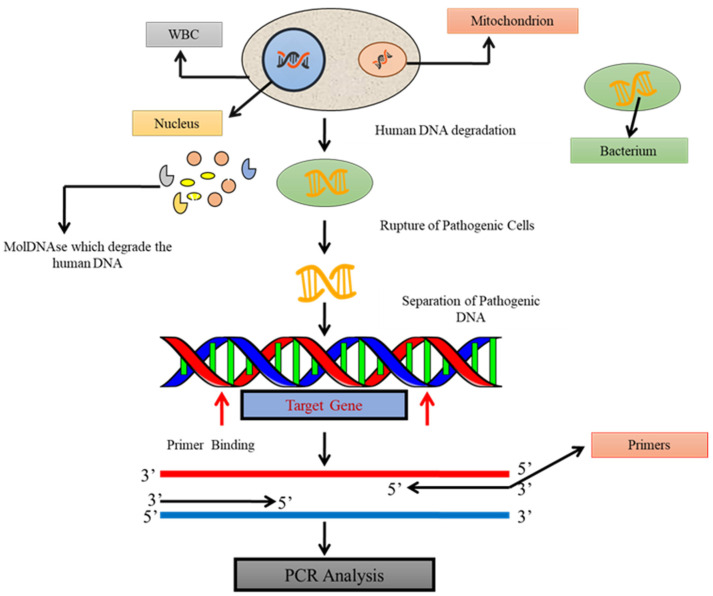
The principle of testing pathogenic DNA by standard PCR in blood samples. Schematic diagram showing the amplification of a segment of DNA, using PCR where the target gene of bacterial DNA is synthesized into two new strands of DNA.

In 1983, Kary Mullis [44] devised the standard PCR, which allows the detection of a single bacterial pathogen by identifying a specific target DNA sequence [45]. A sensitive examination is portrayed here to classify the microorganisms in the entire bloodstream by the PCR as demonstrated in Figure 2. A particular primer–probe set is intended to reproducibly detect bacteria of purified DNA from whole blood. This assay framework was demonstrated to be comprehensive for all strains, equally from all bacteria. This unique PCR-based test was created to assist amplification from a range of human, bacterial and yeast genomic DNAs due to its inefficiency. A broad sample preparation methodology was designed that was applicable for the DNA purification from various bacteria in whole blood. With the help of this method, it was feasible to distinguish every particular bacterial DNA from whole blood samples inoculated with a minimum of 4 CFU/mL. Co-purified human blood DNA did not influence the sensitivity of detection by PCR [46]. PCR can identify even a single copy of a target DNA sequence under ideal conditions in a given sample. Therefore, prior multiplication enrichment of the microbe is not needed, all things considered with basic DNA probe tests. Thus, PCR-based diagnostic tests have made a significant advanced improvement for infectious agents subsequently [47].

The technology has been updated to expand the usage of traditional PCR. The use of the primers pair in parallel reactions with simultaneous amplification for different target DNA sequences, known as multiplex-PCR, is the first absolute shift. As a result, several DNA sequences replicated in the same processor might be amplified [48]. Another change is nested PCR, which uses two sets of primers with a preferred target for amplification of an internal DNA sequence. The first reaction is carried out using the first set of primers, and the results are subsequently subjected to a second amplification with various primer sets [49].

#### 3.1.2. Real-Time PCR

Despite several advances, high-throughput science research laboratories can redirect standard PCR methods, including nested PCR techniques, due to the immense exposure of excess contamination with amplified products [50]. Traditional PCR procedures rely on automated detection of fluorescence from PCR amplicons, while real-time PCR systems are faster, less sensitive to contamination and require less labour [51,52]. Real-time PCR techniques also allow for infinite and comparable calibration of the desired sequence, which may help to increase the gap in critical microbial potential [53,54]. In addition, compared to traditional PCR, real-time PCR has several novel advantages, including simplicity, quantitative capacity and speed [55].

Fluorescence-based real-time PCR is based on the detection of the fluorescent signal generated during DNA amplification. After a specific number of cycles, the real-time assays synthesize a quantity of target DNA. When the amplification of a PCR product is controlled, it is initially observed during cycling by determining the cycle number at which the reporter dye’s discharge energy dominates the background noise. As a result, the threshold cycle is named after this cycle number (Ct). This Ct value is determined during the PCR exponential phase and is inversely proportional to the target’s copy number. As a result, the difference in fluorescence signal is observed before the incident when the beginning of the copy number of the target DNA is higher, and the Ct value is lower [56].

SYBR Green, TaqMan, molecular beacons and scorpions are the four types of fluorescent DNA probes currently available for real-time PCR product detection. All of these probes emit a fluorescent signal that can be used to detect the PCR products mentioned in Table 1 [57]. The ability to quantify the process is a key function of RT-PCR [58]. An internal calibrant is put to each well of the assay plates as a reference in each experiment, and PCR is used to examine each well for quantification. The peak heights in the mass spectrum for amplicons determine the proportionate ratios of calibrant to microorganisms [59,60]. The concentration of the pathogen can be thoroughly evaluated using the known initial concentration of calibrant. Having a significant effect on pathogen detection in diagnostic microbiology, real-time PCR is primarily focused on the pathogen genotype [61]. The role of microbes in various human diseases is examined in these essays, which is the most critical use of real-time technology in microbiology. Due to the strength of complexity the technology has positively evaluated the various microbes and their nucleic acid targets present in a single sample, also it has allowed the differentiation of various forms of microbial genotypes in a single reaction tube [62].

The significance of RT-PCR in microbial load detection is that these techniques are extremely useful since they actively reveal the spectrum of increasing infection, host–pathogen interaction and antimicrobial medication effectiveness. It also enables the administration of antibiotics promptly. Real-time assays are useful for distinguishing serotypes within a particular microbial population [73], diagnosing pathogens in clinical samples [74,75] and bacteria (viruses, bacteria, fungi, protozoa or toxins produced by them that cause diseases) used as biological warfare agents [76]. Despite these, molecular techniques including PCR do not provide any information about AMR among pathogens [77].

#### 3.1.3. Surface-Enhanced Raman Spectroscopy (SERS)

Surface-enhanced Raman spectroscopy (SERS) is emerging as an important technology for the identification which amplifies the Raman dispersing of the objective particles on a superficial layer of metal made of graphene or other different materials [78,79,80,81,82]. This technique has the ability to facilitate the label-free nucleic acid identification [83]. Surface plasmons are generated by applying an excitation frequency that is in phase with the particle’s plasmon assimilation profile, resulting in a solid electromagnetic field on the metal surface. The emission of the Raman dispersed light is radiated in every direction of the particle is gathered through a microscope and consequently identified. In addition, the Raman dispersed light is then coordinated against a reference profile of microorganisms to be recognized [84,85,86,87]. Consequently, SERS can efficiently recognize the occupancy of microbial cells on the outer surface, thus yielding a data-rich spectrum that can be used for microorganism identification [88,89,90,91,92,93,94]. In addition to the pathogen identification, SERS has also been utilized for antibiotic susceptibilities in urosepsis [91]. The primary reason the SERS procedure has not been set up as a routine scientific strategy is that it does not withstand its high sensitivity and specificity, which are the significant disadvantage of SERS, and restricted ability in investigating polymicrobial tests which are because of the lower reproducibility of the SERS signal [95]. Thus, SERS combined with different methods should be incorporated which outlines the wide uses of this incredible method.

#### 3.1.4. MALDI-TOF

Matrix-assisted laser desorption ionization-time of flight mass spectroscopy (MALDI-TOF-MS) is another newly discovered procedure that is now being used in clinical research to detect bacterial species. This technique guarantees fast detection of causative bacterial microorganisms demonstrated to be viable in the positive blood cultures that can rapidly recognize bacterial development, hence accelerating the general process of the antimicrobial resistance report [96]. MALDI is a protein identification ionization technique in which the analyte crystallizes in a strong lattice matrix crystal that absorbs laser light, allowing it to ionize and desorb from the matrix. The ionized atoms are isolated and dependent on their mass to charge (m/z) ratio from the entire microscopic organism’s test when it flies through a vacuum tube produces an m/z profiles of the apparent multitude of proteins in the sample [97]. Mass spectroscopy has been utilized for the inoculation of different species, along with the microbial suspension. Because of the low microbial concentration, MS cannot conduct direct examinations on human blood samples, which is a major disadvantage. Because of the low reproducibility and changeability in preliminary methodology and the matrix composition, blood culture is typically needed to improve the microscopic organisms to a detachable level [98]. Another drawback of MS is its restricted capacity in arranging various microbes from the polymicrobial samples [99,100,101] as the spectral profiles formed by this technique are more complicated, making it difficult to deconvolute the composite spectra gathered at the same time from different microbial species in the poly-microbial samples.

### 3.2. Broad-Spectrum Genomic Detection of AMR

**Figure 3:**
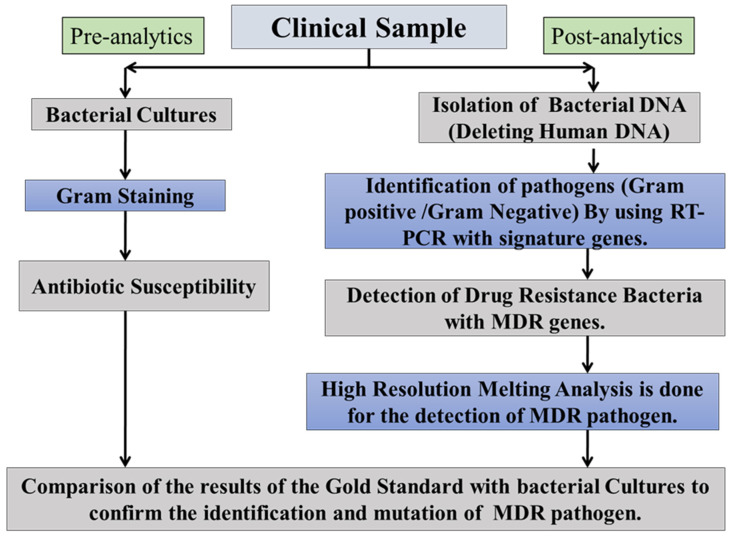
Molecular antimicrobial detecting testing assays. The ideas presented here are relevant to novel antimicrobial testing methods based on nucleic acid amplification. Arrows follow the direction from a clinical specimen to a diagnostic result. A process for the molecular test (right-sided) has been demonstrated, which includes quick sample preparation, amplification and amplicon characterization.

The currently employed technique to detect AMR is time taking (3 to 5 days) and hampers the clinical management of sepsis results in a poor outcome. This protocol is discussed herein Figure 3 for some of the methods. With the advent of genome sequencing, AMR can be detected for both targeted (high resolution melting) and non-targeted (sequencing) pathogens.

#### 3.2.1. High Resolution Melting Analysis Technology

High resolution melt (HRM) analysis depends on the detection of differences in melting temperature (Tm) due to the presence of a mutation in a previously amplified target that produces melt curve profiles specific to pathogens. The size and the sequence of the PCR amplicon is the major reason on which the melting curve profile depends [102]. It is so sensitive that even a single point mutation resulting in a Tm shift can be detected [103]. Therefore, it allows molecular detection of resistant genes and hereditary mutations rapidly with a higher output of post-PCR examination which allows the researchers to identify and classify the new hereditary mutations and variations along with single nucleotide polymorphisms without sequencing (gene scanning) or before sequencing in a population [104]. Several antibiotic resistance marker genes conferring to a bacterium have been usually detected (Table 2). A study reported real-time PCR-based rapid identification of Escherichia coli, Staphylococcus aureus, Enterococcus faecalis and Proteus mirabilis using 16S rRNA gene-specific primers. Furthermore, HRM and machine learning algorithm approaches were used to determine the antimicrobial susceptibility test within 6.5 h [105].

#### 3.2.2. Sequencing

In the diagnostic field, the aim of the utilization of genomic rather than gene-based techniques for both bacterial species detection and AMR detection is growing. There is a need for more effective and rapid AMR preventive measures driving the shift to whole-genome sequencing (WGS). Bacterial and AMR gene identification using automated bioinformatics examination methods are easy to perform after an organism has been isolated by culture [114]. WGS allows all genes involved in resistance to be tracked, allowing all genomic data of resistant factors to be present in a bacterial cell to be analyzed. Next-generation sequencing (NGS), which has revolutionized the biological sciences, is another emerging tool. NGS makes large-scale whole-genome sequencing (WGS) affordable and realistic for the average researcher with its super high throughput, versatility and speed. It also allows scientists to sequence the entire human genome in a single experiment, allowing them to research biological processes at a level never before possible. Furthermore, in the age of complex genomic science, which necessitates a deeper understanding of details outside the boundaries of conventional DNA technology, it has filled the gap and become a routine research method to resolve those issues [115]. NGS combined with meta-genomic approaches that essentially include genome sequencing of infectious biological samples such as blood, urine and lavage without culturing them and provide the diverse profile of all species including those that are targeted and untargeted present in the sample. This approach has revolutionized the identification of all including new resistance genes in a single specimen [116,117]. The sequencing-based metagenomic approach has analyzed the kinetics of gut microbiota before, during and after antibiotic treatment [118]. Furthermore, with the help of machine learning, it is now possible to predict the recolonization of micro-biota post-antibiotic treatment, hence will be helpful in individual patient-specific antibiotic treatment regimens. Expensive and more labour-intensive factors while handling a significant number of samples are the major drawbacks in the wide application of these genome-based methodologies despite their promising applications in pathogen detection [119].

#### 3.2.3. DNA Microarray

Microarray innovation has been utilized for longer than 10 years for the identification of microorganisms which have altogether added to our comprehension of pathogenic mechanisms, microbe reactions to ecological improvements and host–microorganism associations with the immediate effect on diagnostic microbiology [120]. Microarrays have been updated as useful methods for bacterial detection and identification due to their strong parallelism in screening for the expression of a wide variety of genes after specific gene amplification by either a broad-range or a multiplex-PCR before microarray analysis [121] Microarrays employ surface-immobilized DNA and RNA probes to collect and categorize DNA/RNA of microorganisms via sequence-specific complementary hybridization, decreasing sample and reagent consumption and costs while permitting precise segregation down to the species or strain level. A study conducted by Ballarini et al. utilized an oligonucleotide-based microarray (BactoChip) for culture-independent detection, quantification as well as differentiation from 21 different bacterial genera among clinical isolates [122]. Additionally, the Verigene stage from Luminex Corporation can distinguish the Gram-positive board of nine species of bacteria and three genes of AMR against methicillin and vancomycin and five species and six AMR genes for carbapenemase and expanded range beta-lactamases [123,124,125]. Being independent of culture, microarray-based detection is rapid, hence gaining importance in clinics in combination with antimicrobial stewardship [126,127]. Despite these, not a single microarray platform has been commercialized so far to effectively recognize all microorganisms in polymicrobial diseases [128].

## 4. Advanced Methods for Management of Sepsis

Enhancement in the sensitivity and specificity of the current technology with the correct and rapid data analysis in a short time along with identification of drug susceptibility to cumbersome resistance is needed within an hour [129].

### 4.1. Biosensors

Since biosensors have more acknowledged extension for the accurate diagnosis of sepsis, accordingly, headway in this field has given novel world-view progressed highlights. The best methodology for the use of this innovation is higher sensitivity for the diagnosis and identification giving a helping hand to clinicians all over the world to control the disturbing death rate [130]. Because species–specific probes or antibodies give an electrical signal after attaching to their targets, and that signal intensity correlates to the target species total signal, the electrochemical method is the primary criteria on which diagnostic biosensors frequently operate towards identification. They do not just distinguish microbes with an incredible specificity very quickly to hours; however, they also give the data about drug sensitivity [131]. Most biosensors have the constraints in their degree and insufficient for the expansive range recognition as their sensitivity might not efficient enough to distinguish BSI where the pathogenic concentration can be a simple 1–10 CFU/mL in the blood [132] but they offer a quick strategy for analysis of a particular infection or microorganisms utilizing just little sample volumes [131]. More research work is required on biosensors to minimize the expenses and enhance their sensitivity, as the specificity and pace of current advances are unusual and may be useful in critical situations.

### 4.2. Point of Care Test

**Figure 4:**
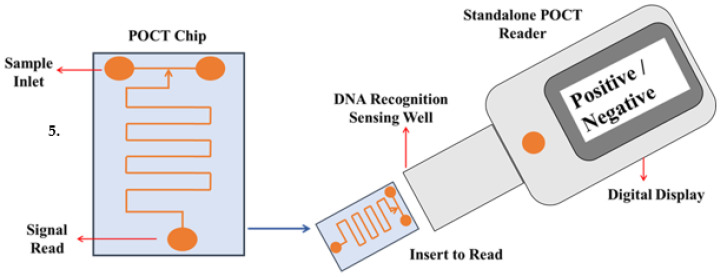
Diagrammatic representation of an ideal POC sensor is shown here which gives the critical information regarding sepsis including both pathogen and host–response information that provides bimolecular identification of the pathogen and quantification of host–response biomarkers.

The restricted advancement which has been previously occurred for the improvement of diagnostics and therapeutics for septicemia is the absence of progress in the immuno responses from sepsis patients has created a challenging part for the advancement of powerful immune therapy along with the proper organization of the anti-infectious agents which activates the dangerous organ dysfunction. Currently, the treatment technique focuses on the administration of anti-toxins, fluid resuscitation and vasopressors [133]. Several studies have shown that early detection of sepsis events and prompt care improves medical outcomes [134,135,136,137]. However, several other studies have also shown that early antibiotic therapy has less significant effect as compared to the control patient cohort, demonstrating the disease’s variability and the need for ongoing testing and treatment [138,139]. As a result, optimal point-of-care sensors allow for the rapid compilation of data related to a patient’s health status, as well as increased health coverage and enhanced healthcare service efficiency while lowering healthcare costs [140,141] (Figure 4). Furthermore, it offers additional pathogen and host–response information practically anywhere with a short processing period, enabling sepsis treatment in two main streams: first, these devices could accelerate the process where optimal care is initiated late, enhancing results, and second, they can quantify multiple entities such as pathogens, plasma proteins and cell-surface proteins, as described as representative of the host immune response, which, when paired with complex data analytics, may help stratify sepsis even at the hospital bedside. Such data could help to expedite the identification of patients who would benefit from the increased treatment [134]. POCT can also potentially diagnose the assessment of the progression of several proteins’ biomarkers (IL-6, IL-10, TNF-a, PCT and CRP) to acute sepsis or septic shock in patients in ICUs, as well as to measure the 28-day risk of all-cause mortality [142] to help with the antibiotic. However, certain POCT parameters, such as temperature regulation and optical signal readout, also necessitate resource-intensive instruments. So far, the research community of POCT has not been informed of the crucial need for polymicrobial infection diagnostics research in this sector, which has been substantially insufficient [143].

### 4.3. CRISPR-Cas9

Advances in biology have been fueled by the technology used to create and modify DNA. The insertion of site-specific changes into the genomes of cells and animals, on the other hand, is still a mystery [144]. CRISPR-Cas9 is a ground-breaking genetic engineering tool that has revolutionized the use of biological RNA-programmable CRISPR-Cas9 in laboratories all over the world [145]. CRISPR/Cas9 is a revolutionary method for editing genomes that is gradually finding applications in numerous domains of biomedical research, including sepsis, as a new way to investigate and cure diseases. CRISPR-Cas9 technology is based on the CRISPR-Cas system, which gives viruses and plasmids in bacteria adaptive immunity. Cas9 is a CRISPR-associated endonuclease that uses the RNA duplex leader sequence tracrRNA:crRNA to base pair with a DNA target sequence, allowing Cas9 to function and induce site-specific double-strand breaks in DNA [146]. The single guide RNA (sgRNA) is designed from the double tracrRNA:crRNA with two key features, i.e., a sequence on the fifth side that determines the DNA target site through base pairing, while a double-stranded RNA structure on the third side connects to Cas9 [147]. Such discovery results in an uncomplicated two-component approach [148] that uses alterations in the Cas9 sgRNA program’s leader sequence to target any DNA sequence of interest [149].

The RNA-guided Cas9 in CRISPR/Cas9 technology for targeted genome editing causes a blunt-ended double-stranded break (DSB) three base pairs upstream of the proto adjacent motif (PAM) domain. To repair DSBs, nonhomologous end joining (NHEJ)- or homology-directed repair (HDR)-mediated disruption or mutation of the genome is utilized where double-strand breaks caused by Cas9 can be repaired in one of two ways. Endogenous DNA repair machinery processes DSB ends before rejoining them via the error-prone NHEJ pathway, which may result in random mutations at the junction point. To take advantage of the HDR pathway, which allows for high fidelity and precise editing, a repair template, such as a plasmid or single-stranded oligodeoxynucleotide (ssODN), can also be given [150]. HDR can also be induced by single-stranded DNA nicks. The unique DNA cleavage mechanism, numerous target recognition capabilities and the presence of many varieties of the CRISPR-Cas system have permitted tremendous improvements in the utilization of this inexpensive and simple-to-operate system-using technology. Genomic loci can be precisely identified, altered, modified, regulated and tagged in a variety of cells and organisms [151,152].

This breakthrough gene editing approach allows for the simultaneous genetic modification of many genes in cells, paving the way for a new class of diagnostics and treatments. This technique is perhaps applied to alter the genomes of contagious microbes as well as host cell genes implicated in the pathogenesis of many illnesses, allowing for better treatment [153]. Furthermore, the potential of the immune system to resist infections can be strengthened using CRISPR/Cas9 technology. Despite advancements in general healthcare, the sepsis high death rate and its repercussions remain unacceptably high. The absence of appropriate therapies is the major cause of this high morbidity and death. Because existing clinical investigations failed to establish wide combination targets, developing creative and evolutionary treatment options to enhance clinical outcomes in sepsis is a primary objective in the prognosis of patients with sepsis [154].

To some extent, sepsis might be considered a hereditary disease, and gene therapy could be a promising new treatment option. In this review work [155], we present a brief application of CRISPR/Cas9 gene editing technology in sepsis research. To begin, here are some guidelines for gene screening: TNF stimulates inflammatory responses while also causing vascular endothelial cell death in sepsis to protect the host from infection. The researchers employed a genome-wide CRISPR/Cas9 knockdown screen to look for potential targets in the TNF signaling system, which controls mild inflammation and apoptosis during sepsis. The benefit of genome-scale screening using CRISPR/Cas9 in sepsis is clearly demonstrated in this work [156]. Second, researchers discovered that mitochondrial DNA (mtDNA) stimulated TLR9 signaling in septic patients, which was probably associated with early inflammation and associated with higher mortality rate in severely ill patients [24]. TLR9 signaling may be triggered by mtDNA, resulting in systemic inflammation, which includes inflammatory mediator overproduction and leukocyte activation [157]. To treat people infected with mutant mtDNA, CRISPR-Cas9 technology can be utilized to delete specific sequences of mutated DNA [158]. The Ccl2 gene product regulates the trafficking of inflammatory monocytes/macrophages, basophils and T lymphocytes in response to inflammatory signals such as TNF- and IL-1 [159]. They modified mouse iPSCs using CRISPR/Cas9 and introduced anti-inflammatory chemicals into the Ccl2 gene. According to the researchers, CRISPR/Cas9 might be used to develop a directed cell-based anticytokine vaccine, which could lead to new pathophysiologic insights and treatments for inflammatory diseases [160].

The therapy options for this condition are currently limited, and employing CRISPR/Cas9 technology to better understand the molecular pathophysiology of sepsis might aid in the development of efficient treatment techniques [161]. Despite the fact that CRISPR/Cas9 revolutionized genome editing, it is far from complete and has several limitations [162]. CRISPR is indeed an RNA-based DNA recognition system that uses an RNA-based guide molecule to detect regions in DNA that have certain molecular features. One of the limitations is that the original Cas9 can only cleave a small portion of the genome if it hits on a genomic region with three “NGG” nucleotide base pairs (N can be any nucleotide). Another drawback is that the Cas9 enzyme only cleaves DNA; approximately 2% of the genome encodes proteins directly from DNA, while the rest, 98%, is the regulatory gene sequence [163]. CRISPR/Cas9 technology may be the simplest and most successful way to perform sepsis-related research; however, due to technical instability and targeting gene constraints, CRISPR/Cas9 research for sepsis therapy requires substantial progress [164].

## 5. Challenges for a New Improved Method

The significant issues confining the fruitful execution of new strategies are the requirement for considerable information, the time it takes to obtain a result, industrial competition and the complexity of the evolving improved methods. In addition, since an experienced individual is needed, these assays can seem difficult to conduct in ordinary microbiology laboratories [165]. Furthermore, the current situation brings benefit to the clinical microbiology lab by allowing for the cost-effective transportation of samples to the lab as well as to the organization of computer-based data results. Nonetheless, the transition time is lengthened by the distance between the laboratory and the time when a sample is collected at the bedside from different hospitals. As a result, laboratories should be prescribed such techniques that prioritize time from sample processing to lab work schedule to patient care, as well as infection prevention steps, for the rapid diagnosis of sepsis. Prior sequencing data for the specific target gene of interest is required for molecular-based approaches. One of the significant drawbacks of molecular processes is that they can only be used to manipulate the identified genes. As a result, accessing the unknown sequence of the gene using some other procedure is an undesirable pitfall of the sequence-based problems that have already been identified [166,167]. When analyzing the data, it is important to remember that the presence of suspected bacteria or DNAemia (the detection of circulating pathogenic DNA responsible for a specific disease) does not always suggest the existence of microbes. Because of the detection of environmental DNA infecting the blood sample or carryover contamination, it might potentially increase the chance of false-positive outcomes. DNAemia is an infection-related condition that can be caused by false septicemia [168,169] or by circulating DNA that persists after many days of successful anti-infectious therapy [170].

Another significant drawback is that they cannot provide any detail on antimicrobial resistance or the detection of the pathogens that may be used to diagnose sepsis. Rapidity in detecting pathogens may allow an excellent and more advanced calibration of this efficient procedure, resulting in greater commercial savings. However, the major issue is the shortcoming of a distinct susceptibility spectrum, particularly with the emergence of multidrug resistant microbes, which could restrict the clinical usage of these assays. Therefore, according to the current situation, it is very important to invest less energy on limited and steady enhancements to existing innovation, yet rather to target for generous improvements so novel approaches with prevalent execution attributes can be affirmed and promoted at the earliest opportunity [171].

## 6. Conclusions and Future Direction

Despite understanding the awareness of the common molecular methods for calibrating gene expression, it is critical to learn about the various choices available in all facets of this science. In comparison to traditional PCR, real-time PCR is much more complex and has a major impact on the final performance. As a consequence, molecular methods can be one of the most responsive and effective methodologies for studying any gene expression analysis in a proper outline carried out with the proper controls [172]. The innovations require enhancements of both pre-analytic and post-analytic updates at the clinical level bringing the issues focused on AMR to more extensive public consideration is an absolute necessity. This will require advancing a superior comprehension of antimicrobial use for reasonable clarifications to the overall population about the drug determination along with the improvement of multidrug resistance from an overdose of antimicrobial agents [173].

With the introduction of novel advancements such as low-cost, high-capacity liquid-handling systems and nucleic acid extraction, modern approaches and innovations are becoming more fundamental and interesting for routine diagnostics. Recent advancements in technology have made it strong and reliable while making it reasonable to challenge easy identification and genotyping with fast reactions and the measurement of a single DNA target following the related quality confirmation programs. There is also a need to improve and verify the full assessment of any newly designed assay against previously used standardized assays, as well as the accuracy of specifications with their appropriate curves. However, recent technology shows that this is a breakthrough that has now arrived [174]. A few promising new techniques and standards are being created and applied in the clinical arena, as we have sketched out in this work, and such upgraded processes will aid and oversee AMR.

In Table 3, we summarize and compare the performance of the technologies in this review in terms of cost, sensitivity, specificity, turnaround time and multiplexing capability of polymicrobial infection diagnosis. Despite progress in technical and clinical improvements, many unanswered questions remain in this sector, necessitating collaboration with not only clinical collaborators, but also regulatory and funding bodies, the diagnostics industry and public health agencies before new approaches such as healthcare systems, skilled nursing and public health agencies can be implemented [175]. Furthermore, a range of advancements will be required to work together, such as the ailments for clinical trials, the use of biomarkers as a technological breakthrough, and these revolutionary developments arise as future directions to focus on a new ground-breaking concept to the diagnosis of sepsis. To keep working on these present diagnostic goals, the prior focus must be on a single hand that is actionable and robust in a well-planned approach to include these pathogen identification results for the treatment of patients with verified disease who will have the desired therapeutic outcomes. As a result, infection to host responses have been selected as the most interesting new technological technique and a viable alternative to pathogen-based diagnoses in the assessment of significant progress [176]. Thus, prospective studies should focus on supervised analysis for correct patient treatment, an integrated “ideal” approach to the diagnosis of sepsis [177,178], as well as labour preparation in wards and labs, which are key ingredients for fruitful programs.

## Figures and Tables

**Table 1:** Comparison of four different types of fluorescent DNA probes currently used in real-time PCR for diagnosing polymicrobial infections.

Fluorescent Molecule	Working	Volume of the Reaction Mixture	Cost-Effective	Sensitivity	Specificity	Sample to Result Time	Detection Limit	Refs.
SYBR Green	Intercalates between the DNA bases to bind to ds-DNA molecules.	10–20 µL	**★★★**	**★**	**★**	2–3 h	60 pg DNA	[63,64]
TaqMan Probes	Taq polymerase performs 5–3 exonuclease activity during hybridization of fluorophore-based detection, cleaving a dual-labeled probe to the corresponding target sequence.	5–10 µL	**★**	**★★**	**★★★**	1–2 h	0.3 pg DNA	[65,66]
Molecular Beacon Probes	The hairpin ring formed by the DNA sequences on the probes ends is designed to be complimentary to one another. The intervening loop portion of the probe is intended to complement the target DNA sequence of interest.	20–25 µL	**★**	**★★**	**★★★**	5–9 h	3–5 pg DNA	[67,68,69]
Scorpion Probes	On the 5′ and 3′ sides of the probe, complementary stem sequences hold a distinct probe sequence in a hairpin loop shape. After the primer is extended during PCR amplification, the identical probe sequence will attach to its compliment inside the same strand of DNA.	20–25 µL	**★**	**★★**	**★★★**	3–5 h	10pg DNA	[70,71,72]

(**★** More stars suggest more specificity and higher sensitivity).

**Table 2:** List of antibiotics classes, resistance marker genes and bacteria used in real-time PCR-HRM.

Antibiotics Class	Genes	Bacteria	Reference
β-lactamases and Cephalosporins	*ctxM*	*Klebsiella pneumonia*	[106]
	*Tem*	*K. pneumonia*, *Pseudomonas aeruginosa*, *Escherichia coli*, *Acinetobacter baumannii*	[106] [107] [104] [108,109]
	*Shv*	*K. pneumonia*	[106]
Fluoroquinolones	*parC*	*Streptococcus* sp.	[109]
	*gyrA*	*Streptococcus* sp.	[109]
Vancomycin	*vanA*	*Staphylococcus aureus*	[110]
	*vanB*	*Enterococcus faecalis*	[111]
	*vanC*	*E. faecalis*	[111]
Methicillin	*mecA*	*S.aureus*	[112]
	*mecC*	*S.aureus*	[113]

**Table 3:** Different infectious polymicrobial diseases: a comparison of molecular diagnostic technologies.

Technology	Cost-Effective	Sensitivity	Specificity	Turnaround Time	Multiplexing Capability	Refs.
Microbiological Methods						
Blood Culture/Gram Staining	**★★★**	**★**	**★**	**★★★**	**★**	[179]
BactecFx/VITEK 2	**★★**	**★★**	**★★**	**★★★**	**★★**	[180]
Biochemical Methods	**★★★**	**★**	**★**	**★★★**	**★**	[181]
Modern Methods						
Molecular Methods						
Real-Time PCR	**★★**	**★★★**	**★★**	**★★★**	**★★**	[182]
SERS	**★★**	**★★**	**★★**	**★★**	**★★**	[183]
MALDI-TOF	**★**	**★**	**★★**	**★**	**★**	[184]
AMR Detection Methods						
HRM	**★★**	**★★**	**★★**	**★★★**	**★**	[185]
Sequencing	**★**	**★★★**	**★★★**	**★**	**★★★**	[173]
DNA Microarray	**★**	**★★**	**★★**	**★★**	**★★★**	[186]
Advanced Methods						
Biosensors	**★**	**★★**	**★★**	**★**	**★★★**	[187]
POCT	**★**	**★★**	**★★**	**★**	**★★★**	[186,187]
CRISPR/Cas9	**★**	**★★**	**★★**	**★★**	**★★★**	[188]
CRISPR/Cas9						

(**★** More stars suggest more cost effective, less sensitive and specificity, more turnaround time and vice-versa according to the technology).

## Data Availability

Data included in the manuscript are available and can be shared if required.
